# Extended photoresponse and multi-band luminescence of ZnO/ZnSe core/shell nanorods

**DOI:** 10.1186/1556-276X-9-31

**Published:** 2014-01-15

**Authors:** Qin Yang, Hua Cai, Zhigao Hu, Zhihua Duan, Xu Yang, Jian Sun, Ning Xu, Jiada Wu

**Affiliations:** 1 Department of Optical Science and Engineering, Fudan University, Shanghai 200433, China; 2 Key Laboratory of Polar Materials and Devices, Ministry of Education, East China Normal University, Shanghai 200241, China; 3 College of Science, Guizhou Minzu University, Guiyang 550025, China

**Keywords:** ZnO/ZnSe core/shell nanorods, Type-II heterojunction, Photoresponse, Near band edge emission, Suppression of luminescence

## Abstract

Aligned ZnO/ZnSe core/shell nanorods (NRs) with type-II energy band alignment were fabricated by pulsed laser deposition of ZnSe on the surfaces of hydrothermally grown ZnO NRs. The obtained ZnO/ZnSe core/shell NRs are composed of wurtzite ZnO cores and zinc blende ZnSe shells. The bare ZnO NRs are capable of emitting strong ultraviolet (UV) near band edge (NBE) emission at 325-nm light excitation, while the ZnSe shells greatly suppress the emission from the ZnO cores. High-temperature processing results in an improvement in the structures of the ZnO cores and the ZnSe shells and significant changes in the optical properties of ZnO/ZnSe core/shell NRs. The fabricated ZnO/ZnSe core/shell NRs show optical properties corresponding to the two excitonic band gaps of wurtzite ZnO and zinc blende ZnSe and the effective band gap between the conduction band minimum of ZnO and the valence band maximum ZnSe. An extended photoresponse much wider than those of the constituting ZnO and ZnSe and a multi-band photoluminescence including the UV NBE emission of ZnO and the blue NBE emission of ZnSe are observed.

## Background

Zinc oxide (ZnO), with a wide band gap (3.37 eV) and a large exciton binding energy (60 meV) at room temperature together with its excellent combined properties [1,2], is regarded as a promising material in a variety of applications, especially in photoelectronics. Because of its high electron mobility and good chemical stability, ZnO has also attracted much attention for photovoltaic applications [3,4]. Various ZnO nanostructures, such as nanorods (NRs) and nanowires in particular, are most promising because their properties can be tailored by changing their morphology, structure and size, or modifying their surface with coatings of other materials [5,6]. Due to its wide band gap, however, ZnO itself can only utilize the light in the ultraviolet (UV) region which accounts for 3% to 5% of the solar energy reaching the earth. Therefore, ZnO has been proposed to form heterojunctions with a narrower band gap semiconductor to extend the spectral region of photoresponse. Zinc selenide (ZnSe), another important Zn-based II−VI semiconductor with a direct band gap of 2.67 eV [7,8] and its good compatibility with ZnO, has been supposed as an ideal material for ZnO to construct heterojunctions [2,9,10].

Aligned ZnO nanorods (NRs) or nanowires are superior to the bulk or film materials in both the surface-to-volume ratio for modifying the surface [9] and the lateral size for reducing the nonradiative recombination and carrier scattering loss [11,12]. The modification of surface and interface has been proved to be one of the most advanced and attractive methods to construct novel nanostructures with tailored properties. The surfaces of ZnO NRs can be decorated with ZnSe coatings, constructing the so-called aligned core/shell type-II heterostructures. Compared with the single constituting materials, heterostructures constructed from such nanostructured ZnO and ZnSe can provide better performance when used in photovoltaic process. The band offset between ZnO and ZnSe together with the resulted effective band gap of ZnO/ZnSe core/shell heterojunctions is favorable for improving the transport of both electrons and holes as well as extending the light absorption region to match the solar spectrum. Meanwhile, the staggered band alignment in type-II heterojunctions facilitates the separation of photogenerated electrons and holes, which is an essential procedure in a photovoltaic device and quite significant to enhance the conversion efficiency of solar cells.

In this work, we studied the optical properties corresponding to the respective excitonic band gaps of wurtzite ZnO and zinc blende ZnSe for ZnO/ZnSe heterojunctions in the form of ZnO/ZnSe core/shell NRs. Aligned virgulate ZnO/ZnSe NRs composed of wurtzite ZnO cores and zinc blende ZnSe shells were fabricated by pulsed laser deposition of ZnSe coatings on the surfaces of hydrothermally grown ZnO NRs. The optical properties of the samples were studied by photoluminescence (PL) measurements which show a significant reduction in the emission from ZnO and co-appearance of the near band edge (NBE) emissions of both ZnO and ZnSe. The former suggests the suppression of radiative recombination of photogenerated carriers, while the latter reveals an extended photoresponse which was further confirmed by optical transparency measurement. Both are favorable for photovoltaic applications.

## Methods

### Sample fabrication

Prior to the growth of ZnO NRs, a dense nanocrystalline ZnO (NC-ZnO) film (approximately 20 nm) was first deposited on a chemically cleaned Si (100) substrate by plasma-assisted pulsed laser deposition. ZnO NRs were grown on the NC-ZnO-seeded Si substrate by hydrothermal reaction. The deposition of NC-ZnO film and the growth of ZnO NRs have been described previously [13]. Serving as the cores, the prepared ZnO NRs were transferred to a vacuum chamber and fixed on a rotating table for the deposition of ZnSe coatings as the shells. The second harmonic of a Q-switched Nd:YAG laser was used to ablate a ZnSe target after being focused by a spherical lens. The laser wavelength, pulse duration, and repetition rate were 532 nm, 5 ns, and 10 Hz, respectively. The focused laser beam with a spot size of 1.2 mm^2^ was incident on the target surface at an angle of 45°. The laser fluence on the target surface was 2 J/cm^2^. ZnSe was deposited at a base pressure of approximately 10^−4^ Pa for 30 min. The deposition of ZnSe coatings were performed at room temperature (RT) or at an elevated temperature of 500°C. The ZnO/ZnSe core/shell NRs obtained by depositing ZnSe at RT were annealed at 500°C in a flowing N_2_ atmosphere (approximately 10^5^ Pa) for 1 h.

In this paper, bare ZnO NRs without ZnSe shells, as-fabricated ZnO/ZnSe core/shell NRs with RT deposition of ZnSe, as-fabricated ZnO/ZnSe core/shell NRs obtained by depositing ZnSe at 500°C, and ZnO/ZnSe core/shell NRs with RT deposition of ZnSe followed by annealing at 500°C in N_2_, are named as samples A, B, C, and D, respectively.

### Characterization and measurements

The sample morphologies were examined by field emission scanning electron microscopy (FESEM) with a Hitachi S-4800 microscope (Dallas, TX, USA). The crystal structures of ZnO and ZnSe in the samples were characterized by X-ray diffraction (XRD) with a Rigaku D/MAX 2550 VB/PC X-ray diffractometer (Shibuya, Tokyo, Japan) using Ni-filtered Cu Kα radiation (*λ* = 0.15406 nm). Fourier-transform infrared (FTIR) spectroscopy and Raman scattering spectroscopy were also used to characterize the structures of ZnO and ZnSe through vibrational mode analysis and phase identification. FTIR spectroscopy was carried out with a Bruker Vertex 80 V spectrometer (Saarbrucken, SL, Germany). Raman measurements were performed with a Jobin-Yvon LabRAM HR 800 UV micro-Raman spectrometer (Villeneuve d'Ascq, France) using a 488-nm Ar^+^ laser beam or 325-nm He-Cd laser beam as the exciting sources. The photoluminescence (PL) of the samples was measured by exciting the samples with 325-nm laser light from a continuous wave He-Cd laser at room temperature to examine the influences of the ZnSe shells on the luminescence from the ZnO cores. The luminescence was detected by an intensified charge-coupled device (ICCD) (iStar DH720, Andor Technology, Belfast, UK) after being dispersed by a 0.5-m spectrometer (Spectra Pro 500i, Acton Research, Acton, MA, USA). The optical properties were also characterized by comparing the optical transparency of ZnO/ZnSe core/shell NRs with that of bare ZnO NRs. The transmission spectra of the bare ZnO NRs and the ZnO/ZnSe core/shell NRs prepared on transparent fused silica plates were measured in the UV-near IR range using a Shimutsu UV3101PC Photo-Spectrometer (Nakagyo, Kyoto, Japan).

## Results and discussion

### Morphology

**Figure 1:**
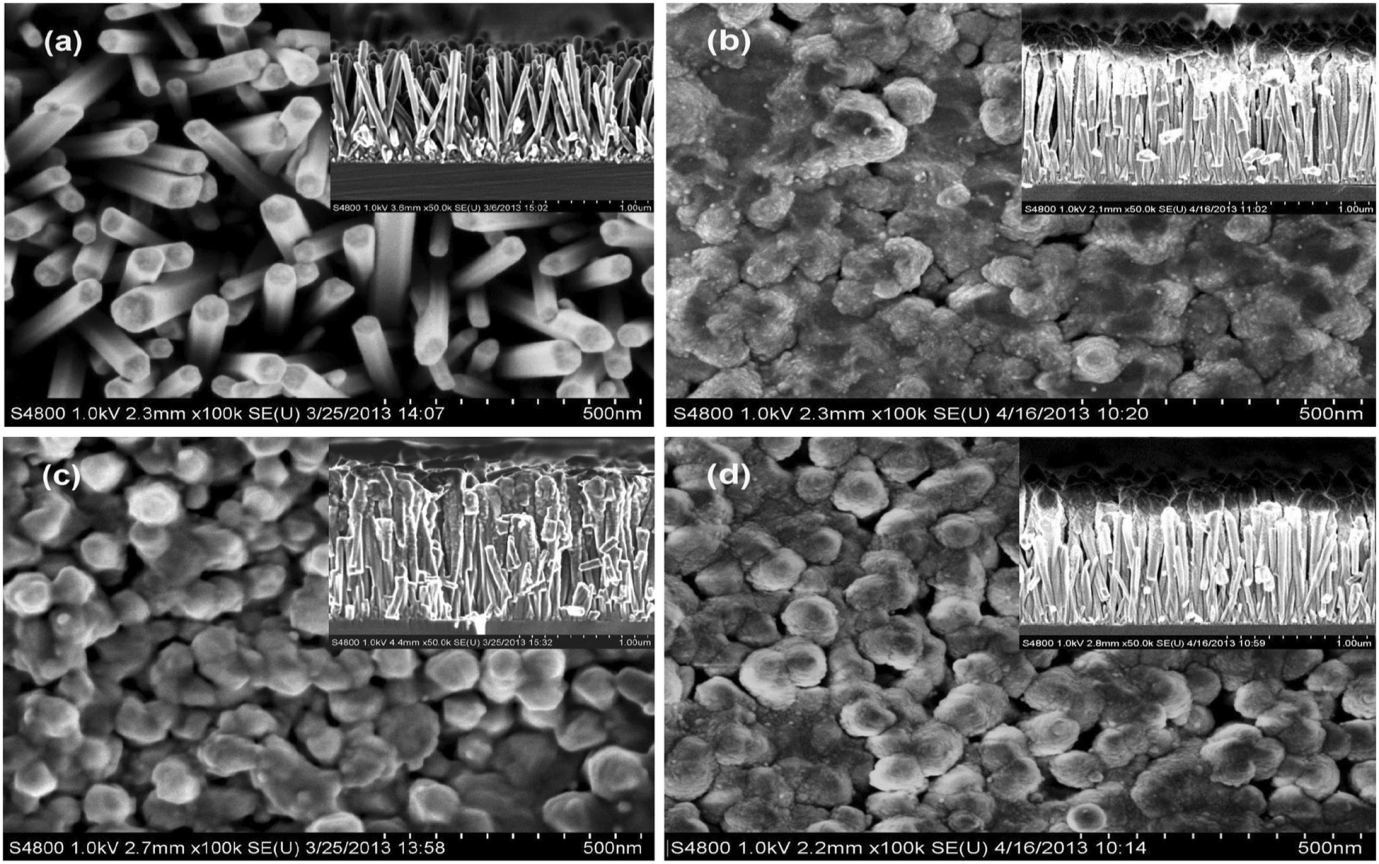
FESEM images showing the top view and cross-sectional view of samples A (a), B (b), C (c), and D (d), respectively.

The FESEM images in Figure 1 illustrate the morphologies of the samples. As shown in Figure 1a for sample A, the bare ZnO NRs grew almost vertically on the substrate, nearly in the shape of hexagonal prisms with a mean diameter of approximately 60 nm and an average length of approximately 1 μm. As will be described below, structural characterization reveals that the hydrothermally grown ZnO NRs are hexagonal wurtzite in crystal structure with preferentially *c*-axis-oriented growth. After the deposition of ZnSe whether at RT or at 500°C, the NRs increase in diameter with rough surfaces (Figure 1b,c), indicating the covering of the ZnO rods with ZnSe shells. However, the NRs in sample B show larger diameters and rougher surfaces than the NRs in sample C. The NRs in sample B are connected together at the rod tips and near the top surfaces, while those in sample C are generally separated from each other from the top to the bottom. In addition, it can be seen from the cross-sectional image of sample B that generally, the diameter at the rod top is slightly larger than that at the rod bottom. This means that when being deposited at RT, ZnSe was more likely to gather on the top surfaces or stack in the upper parts of the gaps between the rods, rather than diffusing smoothly to the bottom. At 500°C, in contrast, ZnSe was uniformly deposited on the whole surface of the ZnO NRs. The deposited ZnSe can diffuse on the side surfaces of ZnO NRs at elevated temperatures to form ZnSe shells outside the ZnO cores. It seems therefore that high-temperature deposition of ZnSe is more suitable for the fabrication of ZnO/ZnSe core/shell NRs than RT deposition. The images of Figure 1d show that sample D has a better morphology than sample B; however, the deposited ZnSe still remains mainly in the upper parts of the gaps. Although the morphology can be improved to a certain extent by high-temperature annealing, the samples prepared by RT deposition of ZnSe followed by annealing are not as good in morphology as those prepared by depositing ZnSe at 500°C.

### Structure

**Figure 2:**
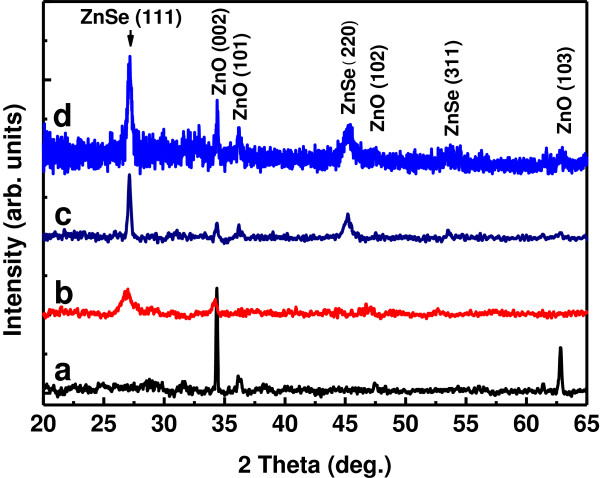
XRD patterns of samples A (a), B (b), C (c), and D (d), respectively.

Figure 2 illustrates the XRD patterns of the obtained samples. The typical XRD pattern of sample A (curve a) is dominated by a narrow peak at 2*θ* = 34.38° with a full width at half-maximum (FWHM) of 0.15°. This peak is indexed to the (002) diffraction of hexagonal wurtzite ZnO (JCPDS: 36–1451). Another distinct peak at 2*θ* = 62.83° and two other weak ones are identified to be diffracted by the (103), (101), and (102) planes, respectively, also indexed to wurtzite ZnO. The bare ZnO NRs are therefore wurtzite with a preferred *c*-axis orientation in crystal structure and present nanocrystalline nature composed of nano-sized crystallites. The lattice constants are calculated to be *a* = 0.321 nm and *c* = 0.522 nm from the XRD data, close to the constants of bulk wurtzite ZnO (JCPDS: 36–1451). And the mean size of the crystallites is estimated to be about 48 nm according to Scherrer's formula [14].

Besides the ZnO (002) peak, the XRD pattern of sample B shows one broad peak located at 2*θ* = 26.86°. This peak is attributed to the (111) diffraction of face-centered cubic (FCC) zinc blende ZnSe (JCPDS: 37–1463). The broadening of the diffraction peak indicates the small crystallite size of the deposited ZnSe. Moreover, the ZnO (002) peak exhibits a small shift (approximately 0.2°) toward the smaller angle side, suggesting that the lattice of the ZnO cores suffers a tensile strain. This can be attributed to the growth of the ZnSe shells outside the ZnO cores since ZnSe has a much larger lattice constant than ZnO [9]. For sample D obtained by annealing sample B at 500°C in N_2_, both the ZnSe (111) and the ZnO (002) peaks show an increased intensity and a narrowed FWHM compared with sample B, indicating an improvement in crystal quality of ZnSe and ZnO due to annealing. Furthermore, two additional peaks are observed at approximately 45.3° and 53.5°, respectively, which can be assigned to the (220) and (311) diffractions of cubic zinc blende ZnSe. The lattice constant of ZnSe is determined to be *a* = 0.568 nm. Contrast to sample B, more diffraction peaks are observed for sample C with the ZnSe (111) diffraction exhibiting a higher intensity and a narrower FWHM, indicating that sample C has a better crystallinity than sample B. The above XRD results suggest that better crystallinity of ZnO cores and ZnSe shells could be obtained either by RT deposition of ZnSe followed by post-deposition annealing or merely by depositing ZnSe at elevated temperatures.

**Figure 3:**
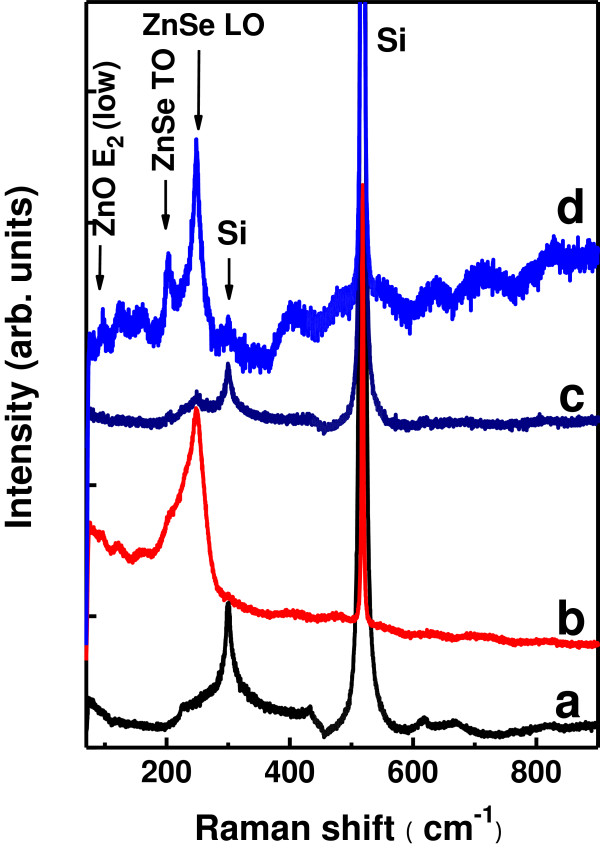
Raman spectra of samples A (a), B (b), C (c), and D (d), recorded by exciting the samples with 488-nm laser beam.

Figure 3 displays the Raman spectra obtained by exciting the samples with 488-nm laser light. For the bare ZnO NRs on Si (100), no distinct peaks related to ZnO are observed besides the signals scattered from the Si (100) substrate. After being deposited with ZnSe shells at room temperature (sample B), the sample scatters a strong and broad peak appearing near 248 cm^−1^ with a FWHM of approximately 31 cm^−1^ (curve b). This Raman scattering corresponds to the longitudinal optical (LO) phonon mode of ZnSe [15-17]. In contrast, the ZnSe LO Raman scattering is much weaker for sample C. ZnSe was uniformly deposited on the side surfaces as well as on the top surfaces of the ZnO NRs, unlike in sample B in which ZnSe was mainly piled up on the top surfaces and in the upper parts of the gaps between the rods. Exciting ZnSe and receiving the scattered light from ZnSe are therefore less efficient for sample C than for sample B. This may be an explanation for the weaker Raman signals scattered from ZnSe recorded for sample C than for sample B. For sample D obtained after annealing sample B at 500°C, the Raman signal attributed to the ZnSe LO mode becomes much narrowed (FWHM approximately 15 cm^−1^). In addition, an obvious peak near approximately 203 cm^−1^ is identified, which belongs to the transverse optical (TO) phonon mode of ZnSe [16-18]. Moreover, a weak but distinct peak at approximately 96 cm^−1^ is observed. This Raman scattering could be attributed to the low-frequency branch of ZnO non-polar optical phonon (E_2_ (low)) [19,20].

**Figure 4:**
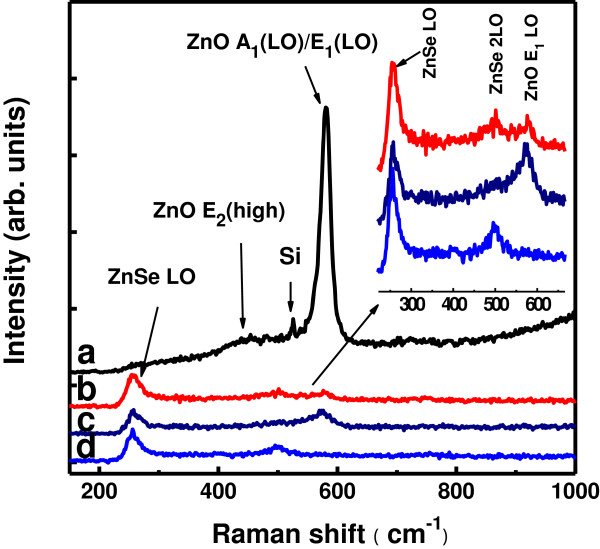
**Raman spectra of samples A (a), B (b), C (c), and D (d), recorded by exciting the samples with 325-nm laser beam.** The inset shows the Raman bands of ZnO/ZnSe core/shell NRs (samples B, C, and D in the downward order).

Raman scattering analysis was also performed by exciting the samples with 325-nm laser light whose photon energy is resonant with the electronic interband transition energy of wurtzite ZnO. The Raman spectrum of sample A is dominated by a Raman peak at 581.5 cm^−1^ (Figure 4, curve a), which corresponds to the LO modes with the A_1_ and the E_1_ symmetries (A_1_ (LO)/E_1_ (LO)) of wurtzite ZnO [21,22], providing an evidence for the wurtzite structure of the ZnO NRs. A weak and broad band centered at 438 cm^−1^ and a sharp peak near 525 cm^−1^ can also be observed. The former is attributed to the ZnO E_2_ (high) mode [20,23] and the latter is scattered from the Si substrate. For the samples of ZnO/ZnSe NRs prepared by depositing ZnSe whether at RT or at 500°C (samples B, C, and D), the ZnSe (LO) mode at approximately 255 cm^−1^ is unambiguously recognized. Furthermore, a weak peak corresponding to the ZnSe 2LO mode at approximately 500 cm^−1^ can also be identified [16,17,21] as shown by the inset in Figure 4. However, the Raman scattering attributed to the ZnO A_1_ (LO)/E_1_ (LO) modes is greatly suppressed due to the ZnSe coatings on the ZnO NRs. The above Raman scattering results obtained with 488- and 325-nm light excitation together confirm not only the wurtzite structure of ZnO cores and the zinc blende structure of ZnSe shells but also the improvement in crystal structures of both the ZnO cores and ZnSe shells by elevated temperature deposition or by post-deposition annealing at elevated temperature.

**Figure 5:**
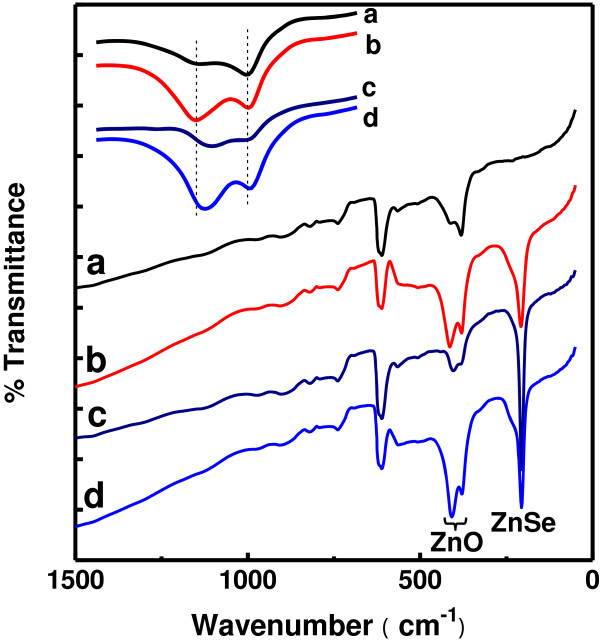
**FTIR transmission spectra recorded for samples A (a), B (b), C (c), and D (d).** The inset shows the position of IR absorption of ZnO in bare ZnO NRs and in ZnO/ZnSe core/shell NRs (curves a, b, c, and d for samples A, B, C, and D, respectively).

The FTIR measurements provide a further evidence for the formation of wurtzite ZnO and zinc blende ZnSe and the influences of deposition temperature and post-deposition annealing. Figure 5 displays the FTIR transmission spectra recorded for the samples. The FTIR transmission spectrum of sample A presents typical characteristics of the IR properties of ZnO. In addition to the absorption of the Si substrate, the principal IR absorption peaks are located in the wavenumber range from 340 to 470 cm^−1^, with one absorption peak near 381 cm^−1^ and another one appearing as a shoulder around 415 cm^−1^. They could be assigned to the stretching modes of Zn − O − Zn. Compared with the bare ZnO NRs, the FTIR spectra of all the ZnO/ZnSe NR samples distinguish themselves with a prominent absorption near 207 cm^−1^ which corresponds to the TO mode of ZnSe [24]. It is also noticed that this absorption peak appears much narrower and stronger for samples C and D, indicating that ZnSe in the samples submitted to high-temperature processing, either depositing ZnSe at 500°C or being annealed at 500°C, has better structure. Also for samples C and D which have experienced high-temperature processing, moreover, the absorption peaks attributed to ZnO exhibit a small red shift, as shown by the inset of Figure 5. These two absorption peaks shift to 378 and 409 cm^−1^, respectively, much close to the ω_T//_ and the ω_T⟂_ frequencies of the ZnO TO modes [25], also indicating that the structure of the ZnO cores was improved during the high-temperature processing.

### Optical properties

**Figure 6:**
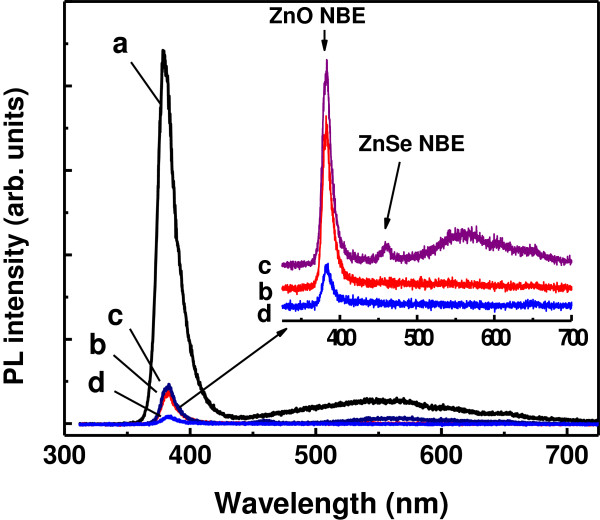
**Room-temperature PL spectra of samples A (a), B (b), C (c), and D (d).** The inset shows magnified PL spectra of ZnO/ZnSe core/shell NRs (curves b, c, and d for samples B, C, and D, respectively).

The bare ZnO NRs are capable of emitting strong and stable UV luminescence (378.9 nm in wavelength, or 3.265 eV in photon energy) when being excited by 325-nm laser light at room temperature, as shown by curve a in Figure 6. This UV emission is associated with the NBE emission of ZnO attributed to the recombination of free excitons [26,27], indicating the high crystal quality of ZnO. The PL spectrum of the ZnO NRs also presents a weak and broad emission band centered at approximately 550 nm (approximately 2.25 eV). This visible emission is usually related to the deep level emission resulted from some defects in ZnO, such as oxygen vacancy, zinc vacancy, interstitial zinc, etc. [28-30]. With the same excitation conditions, all the ZnO/ZnSe core/shell NR samples exhibit weak luminescence, especially the UV NBE emission of ZnO which is greatly suppressed. The suppression of the UV emission is probably due to the quenching of the NBE emission because of charge separation in the heterojunctions composed from ZnO and ZnSe and nonradiative recombination at defect sites in the core/shell interfaces [9,11]. The former is most favorable for photovoltaic application, since the effective charge separation in a type-II heterojunction and the suppressed radiative recombination of photogenerated carriers are highly advantageous to the photovoltaic process. The absorption of the exciting photons in the laser beam and the emitted photons from the ZnO cores by the ZnSe shells could also result in a reduction of the measured luminescence from the ZnO/ZnSe core/shell NRs [9,11]. As will be described later, however, the reduced luminescence measured from the ZnO/ZnSe core/shell NRs could not be attributed to the absorption by the ZnSe shells. It is interesting to notice that for sample C which was prepared by depositing ZnSe coatings on ZnO NRs at 500°C, a distinct emission at approximately 460.5 nm (approximately 2.693 in photon energy) is resolved, as shown in the inset of Figure 6. This blue emission can be attributed to the NBE emission of ZnSe, also associated with free-exciton recombination at room temperature [17,31,32]. In addition, there is a broad emission ranging from 500 to 680 nm in the PL spectrum of sample C. This broad-band emission is seemed to be composed of three bands centered at approximately 530, 617, and 645 nm, respectively. The green emission at about 530 nm and the orange emission at about 617 nm are associated with the vacancies in ZnO [28] and ZnSe [31], respectively. The red emission at about 645 nm could be attributable to the radiative recombination of the electrons in the conduction band minimum of ZnO with the holes in the valence band maximum of ZnSe [9,11].

**Figure 7:**
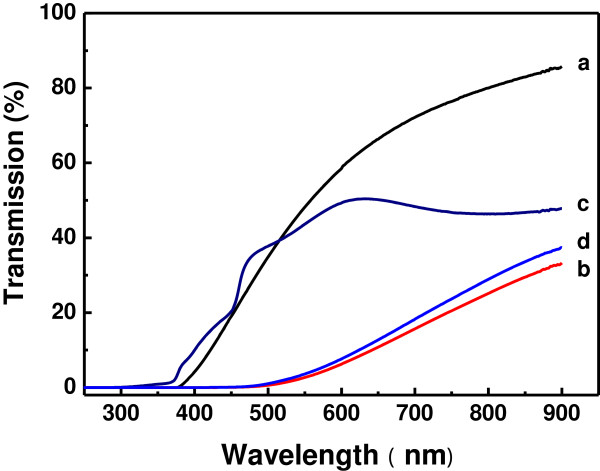
Optical transmission spectra of samples A (a), B (b), C (c), and D (d) fabricated on fused silica substrates.

The transmission spectra of the bare ZnO NRs and the ZnO/ZnSe core/shell NRs prepared on transparent fused silica plates are shown in Figure 7. The samples on fused silica plates were fabricated with the same preparation and post-fabrication processing conditions as used for preparing the samples on Si. The bare ZnO NRs prepared on fused silica exhibit nearly the same morphology, structure, and luminescence as those prepared on Si. The spectra shown in Figure 7 have been corrected for the background from substrate absorption. Unlike ZnO films which usually have high transparency in the spectral region from UV to near IR, the bare ZnO NRs exhibit low transparency with an absorption edge near 380 nm. The low transparency could be attributed to the nano-rod structure in which the incident light will be scattered and trapped. The deposition of ZnSe coatings on ZnO NRs results in a significant decrease in transparency, especially for samples B and D in which the ZnSe coatings were deposited at room temperature. They are almost opaque below 500 nm. It is worthwhile noting the transmission spectrum of sample C which was fabricated by deposition the ZnSe shell coatings on the ZnO rods at 500°C. Though it exhibits lower transparency in the visible region than sample A without ZnSe coatings, its transparency is much higher than samples B and D, indicating that the ZnSe coatings deposited at elevated temperature have better crystal structure and hence better transparency than those deposited at room temperature. It can also be seen that the short wavelength absorption edge shifts to about 370 nm, near the absorption edge of bulk wurtzite ZnO [1], revealing the improvement in crystal structure of ZnO during the high-temperature deposition of ZnSe. The blue shift in the absorption edge and the higher transparency in the short wavelength region of sample C compared with sample A suggest that the reduction in the measured luminescence from ZnO/ZnSe core/shell NRs should not result from the absorption by the ZnSe shells, but from the suppressed radiative recombination of photogenerated electrons and holes because of the enhanced charge separation in the ZnO-ZnSe heterostructures. In addition to the short wavelength absorption edge near 370 nm corresponding the excitonic band gap of 3.35 eV for wurtzite ZnO, another excitonic absorption peak is clearly observed near 460 nm, which corresponds the excitonic band gap of 2.70 eV for zinc blende ZnSe [7,8], also indicating good crystallinity of both ZnO cores and ZnSe shells. These two absorption bands can be correlated with the UV and blue PL emissions, attributed to the respective excitonic band gaps of wurtzite ZnO and zinc blende ZnSe. Moreover, an additional absorption is found extending below the ZnSe band gap into the near infrared. The component below the ZnSe band gap could arise from an interfacial transition coupling a hole state in the ZnSe shell with an electron state in the ZnO core, i.e. the transition corresponding to the so-called effective band gap formed between the conduction band minimum of ZnO and the valence band maximum ZnSe [9,11].

The above optical characterization based on the measurements of transmission spectra and PL spectra reveal that the fabricated ZnO/ZnSe core/shell NRs have a photoresponse much broader than those of the constituting materials ZnO and ZnSe. The extending of photoresponse makes the ZnO/ZnSe core/shell NRs promising as absorbent materials of solar radiation in solar devices.

## Conclusion

In this work, we studied the optical properties of vertically aligned ZnO/ZnSe core/shell NRs after morphology and structure characterization. By pulsed laser deposition of ZnSe on the surfaces of hydrothermally grown ZnO NRs, type-II ZnO/ZnSe heterojunctions constructed of ZnO cores and ZnSe shells were fabricated. The ZnO core NRs grown vertically on the substrates are composed of nanocrystallites with wurtzite structure, while the ZnSe shells, also composed of nanocrystallites, are zinc blende in crystal structure. The structures of both the ZnO cores and the ZnSe shells can be improved by post-fabrication annealing in N_2_. High-temperature deposition of ZnSe has also annealing effects on the structure of the ZnO cores. At room temperature, the ZnO NRs exhibit a good behavior on UV NBE emission with a weak defect-related visible emission, whereas only a weak PL is observed from the ZnO/ZnSe core/shell NRs because of the suppression of the emission from ZnO cores by the ZnSe shells. The ZnO/ZnSe core/shell NRs fabricated by depositing ZnSe at elevated temperatures are superior to the samples fabricated by depositing ZnSe at room temperature both in structure and optical properties. Multi-band luminescence including the UV NBE emission of ZnO and the blue NBE emission of ZnSe is observed from the samples fabricated by depositing ZnSe at 500°C on the hydrothermally grown ZnO NRs. In addition, the ZnO/ZnSe core/shell NRs fabricated with the deposition of ZnSe at 500°C show an extended photoresponse much broader than those of the constituting ZnO and ZnSe.

## Competing interests

The authors declare that they have no competing interests.

## Authors’ contributions

QY prepared all the samples, performed FESEM, XRD and transmission measurements, and drafted the manuscript. HC measured the PL spectra and participated in manuscript writing. ZGH contributed to the mechanism discussion. ZHD measured the Raman and FTIR spectra. XY participated in the preparation of some samples. JS and NX characterized the sample structure and analyzed the optical properties. JDW designed the research and wrote the manuscript. All authors read and approved the final manuscript.
